# Cerebral Malaria in a Patient with Recent Travel to the Congo Presenting with Delirium: A Case Report

**DOI:** 10.5811/cpcem.2020.8.47995

**Published:** 2020-10-19

**Authors:** Megan T. Roberson, Austin T. Smith

**Affiliations:** Vanderbilt University Medical Center, Department of Emergency Medicine, Nashville, Tennessee

**Keywords:** Malaria, cerebral malaria, fever in returned traveler, Plasmodium falciparum, delirium, altered mental status, travel history, infectious disease, tropical medicine

## Abstract

**Introduction:**

Cerebral malaria, a syndrome of altered consciousness, is a rare and severe neurologic complication resulting from *Plasmodium falciparum*.^1^ Historically, cerebral malaria has been seen more frequently in children rather than adults. To complicate the diagnosis, cerebral malaria has few specific symptoms and neurologic findings can vary with each case.

**Case Report:**

We describe a case of a 61-year-old male who returned from the Democratic Republic of Congo and presented to the emergency department with dehydration, fatigue, and intermittent confusion. He was ultimately diagnosed with cerebral malaria caused by *P. falciparum*.

**Conclusion:**

Even with close monitoring and appropriate treatment, cerebral malaria carries a severe risk of long-term neurocognitive deficits and a high mortality rate.

## INTRODUCTION

Cerebral malaria is an uncommon disease process but should be considered in any patient presenting with neurologic complaints and recent travel to a malaria-endemic area. Most cases of malaria in developed countries are seen in travelers returning from malaria-endemic countries. The World Health Organization (WHO) estimated 228 million cases of malaria worldwide in 2018.^2^ Of those cases 93% occurred in Africa with the Democratic Republic of Congo (DRC) accounting for 12% of all malaria cases worldwide, second only to Nigeria.^2^ It is estimated there are 57 cases of malaria per 1000 population at risk worldwide.^2^ Cerebral malaria is uncommon, especially in the United States (US). Further, the majority of the literature on cerebral malaria is focused on the pediatric populations as it is much more common in children.^3^

We present a case of a 61-year-old male who presented to our emergency department (ED) with diarrhea, fatigue, and delirium after return from the DRC two days prior. He was diagnosed with cerebral malaria and transferred to the intensive care unit (ICU) where he was successfully treated and ultimately left the hospital with no neurologic deficits.

## CASE REPORT

A 61-year-old male with a past medical history of hypertension presented to our ED with a chief complaint of fatigue. The patient had been visiting friends and relatives in the DRC and had returned to the US two days prior to presentation. History was obtained primarily from the patient’s son as his father was delirious, which limited his ability to provide a comprehensive history. The son stated that the patient’s symptoms began one week prior to presentation while still in the DRC. The patient returned to the US in a feeble state per the son. He had very little oral intake over the prior week and would wake up confused and disoriented during the three days prior, including not recognizing his own house or shower on the day of presentation. The patient’s confusion would wax and wane but seemed worse upon wakening in the morning and after napping, and would improve throughout the day. He reported night sweats and chills but no known fevers. He had multiple episodes of dark diarrhea, but no bright red blood or black stools. He had a mild cough but no dyspnea or chest pain. He also reported nausea without vomiting.

A review of systems was otherwise negative including absence of headache, vision changes, numbness, focal weakness, or skin eruptions. The patient did endorse multiple mosquito bites but had been on malaria prophylaxis with doxycycline, which he had continued to take upon his return to the US. The patient’s compliance with medication along with other precautions and malaria preventative measures such as mosquito nets and permethrin spray was not determined in the ED due to the patient’s critical illness and need for resuscitation. He visited the international travel clinic prior to his trip and had been vaccinated for both yellow fever and typhoid. He was unaware of any sick contacts.

On arrival, his vital signs were significant for hypotension with a blood pressure of 103/62 millimeters mercury, tachycardia with a pulse of 123 beats per minute, an oral temperature of 100.3 degrees Fahrenheit with oxygen saturation 98% on room air. Differential diagnosis included meningitis, Ebola virus disease (EVD), chikungunya, enteric fever, filariasis, tick-borne rickettsiae, schistosomiasis, dengue, yellow fever, and malaria. An immediate consult to infectious disease (ID) was placed due to the concern for recent travel to an EVD-endemic area. The ID service determined that the patient was not in a place that had known active EVD at the time; therefore, EVD protocol was not initiated. An electrocardiogram (ECG) was obtained, which showed sinus tachycardia with a QTc interval of 369 milliseconds (msec).

His physical exam was notable only for lethargy. He was slow to answer questions but would answer appropriately. He had no focal neurologic deficits and was alert and oriented to self, place and year, but not to month. He had no neck pain, neck stiffness, jaundice, scleral icterus, or skin eruption.

Laboratory evaluation was significant for a normal white blood cell count with thrombocytopenia (platelets 111 per microliter ]K/mcL] [reference range 135–371 K/mcL]) and anemia (hemoglobin nine grams per deciliter [g/dL] [reference range 14.0–18.1 g/dL], and hematocrit 25% (reference range 41–49%). His serum sodium was slightly low at 131 millimoles per liter (mmol/L) (reference range 136–145 mmol/L) with otherwise normal electrolytes and a normal serum creatinine. Glucose was 177 milligrams/dL (mg/dL) (reference range 70–99 mg/dL). His alanine aminotransferase and aspartate aminotransferase were elevated at 228 units (U/L) (reference range 5–55 U/L) and 125 U/L (reference range 5–40 U/L), respectively. Total bilirubin was elevated at 3.1 milligrams per deciliter (mg/dL) (reference range 0.2–1.2mg/dL), as well as direct bilirubin a 1.3 mg/dL (reference range 0.0–0.5 mg/dL). His serum lactate was 4.9 millimoles (mmol)/L (reference range 0.5–2.2 mmol/L), lactate dehydrogenase elevated at 964 U/L (reference range 125–220 U/L) with a low haptoglobin and a normal prothrombin time test. Urinalysis was without signs of infection. A peripheral blood smear was obtained, which showed malarial parasites present concerning for *Plasmodium falciparum* infection with an initial parasite load of 10%. Multiple (between one and three) intra-erythrocyte rings were seen within single erythrocytes, which are consistent with malaria and was confirmed by ID.

CPC-EM Capsule

What do we already know about this clinical entity?

*Cerebral malaria is a rare, poorly understood complication of* Plasmodium falciparum *that carries significant morbidity and mortality*.

What makes this presentation of disease reportable?

*Cerebral malaria is rare and not commonly seen in the United States*.

What is the major learning point?

*Cerebral malaria should be considered in any patient presenting with neurologic symptoms with recent travel to malaria endemic areas*.

How might this improve emergency medicine practice?

*Prompt recognition and treatment of this deadly disease is important to reducing the morbidity and mortality associated with this disease*.

The recommendations of the ID consultant were doxycycline and intravenous (IV) quinidine, and the patient was admitted to the ICU for continuous IV therapy and closer monitoring of his mental status as well as for the need for frequent laboratory draws and ECGs. IV quinidine can cause significant hyperinsulinemia and resultant hypoglycemia; therefore, the patient received hourly glucose checks. In addition, quinidine can prolong the QT interval, and the patient required hourly ECGs with ID recommendations of stopping quinidine if QTc increased by greater than 50% of patient’s initial baseline and holding the medication until the QTc fell to less than 25% above the original value.

The patient continued to have waxing and waning levels of consciousness and required 24 hours of IV quinidine after which his peripheral smear showed a malarial burden of 0.7%, and he was transitioned to oral quinine. He did not have episodes of hypoglycemia and his longest QTc interval was 540 msec. The patient’s mental status slowly improved as his malarial burden decreased. He required three days in the ICU and was then transferred to the floor. He completed a three-day course of oral quinine while in the hospital, and his final smear had a parasite load of 0%. The patient was discharged on hospital day five on oral doxycycline to finish a total seven-day course. He followed up in the ID clinic two weeks after discharge and had no sequelae. His mental status had returned to normal, he had had no fevers, and had begun to regain his strength.

## DISCUSSION

The definition of severe malaria constitutes a multitude of symptoms including altered level of consciousness, respiratory distress, shock, acute kidney injury, convulsions and others, but has one unifying factor in that it can only be caused by the *P. falciparum* species and is associated with a high mortality rate (greater than 5%) even with appropriate treatment.^4^ Cerebral malaria is a subset of severe malaria and is associated with an impaired level of consciousness. It is difficult to diagnose as it has no specific pathognomonic features to differentiate it from non-cerebral malaria as other malarial species can alter consciousness by separate pathophysiological processes.^4^ These include metabolic abnormalities or systemic effects of infection such as high fever.^4^ WHO characterizes cerebral malaria by unarousable or deep coma with asexual forms of *P. falciparum* parasites on peripheral blood smears and no other cause to explain the altered level of consciousness.^5^ WHO further reported that most published studies use the term cerebral malaria when referring to the syndrome in which altered consciousness, associated with a malarial infection, cannot be attributed to a non-malarial cause. This describes our patient as he was not comatose on arrival to the ED.^6^ As of yet, there has been no agreed upon definition for cerebral malaria, and in practice any patient with altered level of consciousness or other signs of neurologic impairment should be treated as cerebral malaria.

Our patient was from the DRC and had been there visiting friends and relatives (VFR), which is important to note as studies have shown that VFRs are at higher risk for diseases that are largely preventable, including malaria.^7^ The reason for this is multifactorial and includes barriers to preventative medical care prior to travel as well as patient misconceptions regarding their risk.^7^ VFRs in the US have higher levels of poverty, lower levels of formal education, and lower insurance coverage, which leads to less preventative pre-travel medical services.^7^ In addition, many VFRs are thought to perceive less personal risk secondary to their cultural and geographic familiarity with the area, as well as an idea that diseases such as malaria are “normal” or “expected” in the country they are visiting.^7^

In addition to the difficulty in diagnosing cerebral malaria, the treatment carries its own risks. Quinidine has been associated with torsades de pointes related to prolongation of the QT interval as well as hypoglycemia secondary to hyperinsulinemia.^8^,^9^ Because of this, frequent ECGs must be performed and labs drawn, and the patient’s neurologic status must be closely monitored. Another treatment option is exchange transfusion, which has been used in cases of severe malaria, in particular for patients with a parasite load greater than 10%.^10^ Exchange transfusion has been shown to improve laboratory values; however, it has not been shown to increase parasite clearance, ICU and hospital length of stay, in-hospital mortality, and cost of hospitalization.^10^ Our patient had a parasite load of 10% and could have potentially been treated with exchange transfusion as well as the other treatments above; however, ID did not feel this was warranted and exchange transfusion was not pursued.

## CONCLUSION

Cerebral malaria is a leading cause of malaria mortality worldwide, accounting for 20% of adult deaths and 15% of childhood deaths.^11^ Even with appropriate treatment early in the disease course, patients still have a high risk of death and up to 25% of survivors will have neurologic sequelae.^11^ While many factors contribute to a patient’s prognosis, including end-organ damage and severity of neurologic dysfunction, treatment must be given as early as possible to maximize the chance of recovery.^6^ To diagnose this rare disease process early in its course, emergency physicians should be aware of the symptoms and the need for screening in patients returning from a malaria-endemic area with altered levels of consciousness.
